# Oritavancin for the Treatment of *Staphylococcus aureus* Bacteremia—A Retrospective Single-arm Cohort Study

**DOI:** 10.1093/ofid/ofaf333

**Published:** 2025-06-12

**Authors:** H Jordan, K Kozierowski, R Pickles, J S Davis

**Affiliations:** Department of Infectious Diseases, John Hunter Hospital, Newcastle, Australia; Pharmacy Department, John Hunter Hospital, Newcastle, Australia; Department of Infectious Diseases, John Hunter Hospital, Newcastle, Australia; School of Medicine and Public Health, University of Newcastle, Newcastle, Australia; Department of Infectious Diseases, John Hunter Hospital, Newcastle, Australia; School of Medicine and Public Health, University of Newcastle, Newcastle, Australia

**Keywords:** endocarditis, long-acting glycopeptides, osteomyelitis, people who inject drugs, Staphylococcus aureus

## Abstract

**Introduction:**

Patients who require prolonged intravenous antibiotics for *Staphylococcus aureus* bacteremia (SAB) and who are ineligible for outpatient parental antibiotic therapy (OPAT) present a challenge in regard to delivering appropriate and cost-effective treatment. Oritavancin, a novel long-acting lipoglycopeptide, shows promise as an alternative to OPAT for patients with complex Gram-positive infections, including those with SAB.

**Objectives:**

To assess the outcomes, including safety and impact on length of stay and cost-effectiveness of using oritavancin for patients with SAB who are ineligible for OPAT.

**Methods:**

Retrospective single-arm cohort study of adult patients with SAB who received at least 1 dose of oritavancin within the Hunter New England Local Health District between January 2020 and July 2023.

**Results:**

A total of 27 patients were identified: mean age, 43 years, 52% male, and 59% people who currently inject drugs. Sources/foci of infection included bone/joint infection (26%), skin/soft-tissue infection (15%), infective endocarditis (26%), catheter-associated bacteremia (7%), and bacteremia with no clear source/focus (26%). Intravenous antibiotics were administered for a mean duration of 10 days before oritavancin therapy. Clinical cure was seen in 26/27 (96%) of patients, with 1 patient lost to follow-up and deemed not clinically evaluable. Twenty-four of 27 (89%) patients were alive at the end of the 180-day follow-up period, with 2 patient deaths unrelated to their index infection and 1 patient not clinically evaluable. A total of 89% of patients demonstrated a positive return on investment, with on average 18 hospital days per patient avoided.

**Conclusions:**

Oritavancin is promising as a suitable and potentially cost-effective alternative for patients with SAB who are ineligible for OPAT. Prospective studies are required to confirm its utility in clinical practice, in particular in patients who do not receive oral antibiotic stepdown therapy.

## BACKGROUND

*Staphylococcus aureus* is a leading cause of bacteremia, and despite improvements in management, mortality, and hospital-related expenditure associated with *S aureus* bacteremia (SAB) remain high [1]. Although the dogma around the need for prolonged or exclusive intravenous therapy is changing, consensus guidelines currently still recommend intravenous-only therapy for at least 2 weeks for *S aureus* bacteremia [2], and it remains to be proven if early oral switch strategies are as safe and effective as the standard approach of exclusive prolonged intravenous therapy for SAB. Outpatient parenteral antibiotic therapy (OPAT) services are often used to administer prolonged courses of intravenous therapy and subsequently avoid need for ongoing hospitalization; however, OPAT comes with a significant resource burden, is subject to patient suitability, and in itself carries risks of vascular access–related morbidity. Patient groups who are often considered ineligible for OPAT include people who inject drugs (PWID), those without suitable housing, or those who reside in residential aged care facilities; these patients present a challenge, often requiring prolonged inpatient stays for intravenous therapy or choosing to leave the hospital before treatment is completed [3].

Oritavancin is a novel long-acting lipoglycopeptide (LaLGP) with a terminal half-life of 393 hours that has a broad spectrum of activity against Gram-positive organisms including methicillin-sensitive *S aureus*, methicillin-resistant *S aureus*, and vancomycin-resistant enterococci [4]. Structurally an analogue of vancomycin, oritavancin contains an additional substituted sugar and an aromatic lipophilic side chain, giving it both hydrophobic and lipophilic groups [4]. Its prolonged terminal half-life is the result of extensive tissue distribution and slow elimination from tissue sites, with <5% detected in urine 7 days after administration [4]. Although only currently registered for use in treating acute bacterial skin and skin structure infections (ABSSSIs), oritavancin's combined attributes of anti-staphylococcal activity and prolonged terminal half-life make it a promising alternative to OPAT for patients with SAB, particularly those who are ineligible for OPAT [5].

Evidence for the use of oritavancin beyond the indication of ABSSSI is limited to a small number of retrospective observational studies that have looked at its utility in different complex Gram-positive infections [5–9]. Of these, Ahiskali and colleagues included 12/24 patients with SAB, 3 of whom demonstrated clinical failure with oritavancin. All prior studies have included multiple Gram-positive pathogens and, to our knowledge, this is the largest single-center, retrospective, observational study describing the real-world use, clinical outcomes, adverse events, and cost-effectiveness for patients treated with oritavancin for SAB.

## METHODS

This was a retrospective, single-arm, observational study including all patients who received at least 1 dose of oritavancin from January 2020 until July 2023 within HNELHD, a health network including a major metropolitan tertiary referral hospital and several large regional centers, in New South Wales, Australia. Other inclusion criteria were: (1) positive blood cultures for *S aureus* within 42 days before the dose of oritavancin; (2) age ≥ 18 years; and (3) administration of oritavancin (ie, not only dispensed).

An infectious diseases registrar and pharmacist conducted an independent review of the medical record for each event of oritavancin administration. Patient demographics, clinical indication, relevant microbiological results, and details of previous antibiotic treatment including duration were collected. The rationale for oritavancin use was reviewed and categorized based on reason for ineligibility for OPAT—including current injecting drug use or other substance misuse, absence of fixed residential address and other barriers to OPAT including mobility, transportation, or residence in an aged care facility.

Patient outcomes were dichotomously categorized as clinical cure or failure, with clinical cure defined as resolution of all clinical signs and symptoms of infection, with no infection-related readmission to hospital or requirement for recommencement of intravenous antibiotics for the indication initially treated with oritavancin, within the 180 days after the administration of oritavancin. Patients who did not attend formal follow-up appointments but who were indirectly followed up through other health system encounters (including subsequent emergency department attendance and drug and alcohol clinic), without documented concern for relapse of infection, were classified as clinically cured. Patients who did not have any local health system follow-up of any nature were classified as not clinically evaluable. Durations of oral antibiotics provided on discharge were obtained by searching pharmacy dispensing records and national electronic health records, where available, for each patient. A simplified cost-of-healthcare analysis was conducted to evaluate hospital days avoided and assess overall cost effectiveness of oritavancin therapy. Hospital days avoided was calculated by subtracting the actual length of stay (LOS) from the projected length of parenteral treatment. The projected length of treatment was determined by the total inpatient days required for completion of parenteral standard of care (SOC) therapy, as determined by an infectious diseases physician or consensus guidelines. The cost-of-healthcare for patients who received oritavancin to facilitate discharge was calculated as the cost associated with actual LOS plus oritavancin acquisition costs. A cost of a single-day hospital admission to a general ward bed for intravenous therapy was set at $1100 Australian dollars (AUD), as provided by hospital finance officers. This cost estimate excluded the cost of antibiotic acquisition for SOC therapy because the cost of standard antimicrobial therapy in most instances was considered negligible. The average wholesale price of $6000 AUD, as recorded in the hospital pharmacy inventory system in 2023, was used for oritavancin cost (as recorded in the hospital pharmacy inventory system). A positive return on investment (ROI) occurred when the cost of hospital days avoided exceeded the combined cost of actual hospitalization and administration of oritavancin.

A separate cost-of-healthcare analysis was conducted to estimate the theoretical cost difference between inpatient administration of oritavancin before discharge, against the projected cost of admission to the local OPAT service for completion of SOC therapy. The cost of admission to an OPAT service for a single day was estimated to be $340 AUD, comprising $250 AUD in admission costs (as provided by hospital finance officers) plus an additional $90 AUD for antimicrobial therapy, estimated from average drug acquisition costs (obtained from the hospital pharmacy dispensing and inventory system). The cost of admission to OPAT was determined to be equal to the cost of actual hospital admission (actual LOS, in days, multiplied by $1100 AUD) plus the number of hospital days of avoided cost as an admission to OPAT services (hospital days avoided multiplied by $340 AUD).

### Ethics

This study was approved by the Hunter New England Local Health District Human Research Ethics Committee. A waiver of patient informed consent was granted given the retrospective nature of this study.

## RESULTS

Thirty-seven patients had oritavancin dispensed between January 2020 and July 2023, 1 patient was excluded for having the drug dispensed but not administered, and 9 patients were excluded for not having a confirmed SAB. Hence, the study population included 27 patients receiving 30 doses of oritavancin (range of doses per patient, 1–2), their characteristics are outlined in Table 1. All patients received a standard dose of 1200 mg infused intravenously over 3 hours. SAB was confirmed for all patients by the presence of blood cultures growing *S aureus*, with 33.3% (n = 9) being penicillin-susceptible *Staphylococcus aureus*, 48.1% (n = 13) methicillin-susceptible *S aureus,* and 18.5% (n = 5) MRSA. The most common foci of infection were bone/joint infections (n = 7, 26%), infective endocarditis (n = 7, 26%) and bacteremia with no clear focus (n = 7, 26%).

**Table 1.** Baseline Characteristics (N = 27)

Demographics	
Age (y), mean (SD)	43 (14)
Weight (kg), mean (SD)	64 (24)
Gender—male, n (%)	14 (52)
Gender—female, n (%)	13 (48)
Aboriginal/Torres Strait Islander, n (%)	9 (33)
Smoker (current), n (%)	21 (78)
Episode of previous SAB, n (%)	7 (26)
Diabetes, n (%)	3 (11)
Clinical indication	
Bone/joint infection—6 (22)	…
	Septic arthritis—1
	Osteomyelitis—4
	Fracture fixation device infection -1
Soft tissue—4 (15)	…
	Cellulitis—1
	Abscess—3
Infective endocarditis—7 (26)	…
	Native—7
	Prosthetic—0
Line-related bacteremia—2 (7)	…
Bacteremia, no clear source or focus—8 (30)	…
Reasons for oritavancin use	
Indication	N (%)
Current PWID	16 (59)
No fixed address	1 (4)
Nursing home resident	1 (4)
Difficult venous access	4 (15)
Unable to attend HITH (mobility, transportation)	4 (15)
Concerns for compliance	15 (57)

Abbreviations: HITH, hospital in the home; PWID, people who inject drugs; SAB, *Staphylococcus aureus* bacteremia; SD, standard deviation.

Regarding reasons for use of oritavancin, current intravenous drug use was the most common indication (n = 16, 59%), with other nonmutually exclusive indications including clinician concern for patient compliance with OPAT (n = 15, 56%), difficult venous access (n = 4, 15%), and issues with mobility or transportation (n = 4, 15%). Before the administration of oritavancin, intravenous antibiotics were prescribed and administered in all 27 (100%) patients, with a mean duration of 10 days (standard deviation, 8 days; range, 1–31 days). The most commonly used agents were flucloxacillin (n = 18, 67%), cefazolin (n = 5, 19%), and vancomycin (n = 9, 33%). The average length of inpatient stay was 9 days (standard deviation, 8 days; range, 1–26 days) and 12/27 (44%) patients were dispensed oral antibiotics on discharge (ie, in addition to having received oritavancin). Of the 12 patients, 1 (8%) received ≤1 week, 7 (58%) received ≤2 weeks, 2 (17%) received ≤4 weeks, and 2 (17%) received ≥12 weeks; oral antibiotics were commenced immediately after the administration of oritavancin. Details of regimens used are described in Table 2.

**Table 2.** Summary of Indications and Treatment Details

Patient	Age (y)	Susceptibility	Clinical Indication	Details	Duration of Previous IV Therapy (d)	Doses of Oritavancin Administered	PO Antibiotics Given on Discharge	Duration of Antibiotics on Discharge (wk)	Agent/Dose
1	39	MSSA	Bone/joint	Native joint septic arthritis	9	1	No	0	…
2	80	MSSA	Bacteremia, no focus	…	2	1	No	0	…
3	56	MSSA	NV infective endocarditis	…	14	2	No	0	…
4	66	MSSA	Bacteremia, no focus	…	5	1	No	0	…
5	47	PSSA	Bone/joint	T5-S1 level epidural collection + L5-S1 discitis	5	1	Yes	1	Amoxicillin 1 g TDS
6	49	PSSA	Bone/joint	…	0	1	No	0	…
7	62	MSSA	Bacteremia (line related)	…	3	2	No	0	…
9	41	MSSA	NV infective endocarditis	Bilateral L4/L5 + right L5/S1 facet joint septic arthritis with epidural abscess + tricuspid valve infective endocarditis	24	1	Yes	≥12	Dicloxacillin 1 g QID
10	37	MRSA	Soft tissue	…	5	2	Yes	2	Ciprofloxacin 750 mg BD + doxycycline 100 mg BD
11	32	MSSA	Bone/joint	L5/S1 discitis/OM	10	1	No	0	…
12	45	PSSA	Bacteremia (line related)	…	1	1	Yes	2	Amoxicillin 1 g TDS
13	36	PSSA	PV infective endocarditis	…	6	1	No	0	…
14	35	PSSA	Bone/joint	T6/T7 vertebral osteomyelitis/discitis	12	1	Yes	2	Dicloxacillin 1 g QID
15	13	PSSA	Bacteremia, no focus	…	1	1	No	0	…
16	35	PSSA	NV infective endocarditis	…	20	1	No	…	…
17	36	MSSA	Bacteremia, no focus	…	1	1	Yes	2	Dicloxacillin 500 mg QID
18	35	PSSA	Bacteremia, no focus	…	5	1	No	0	…
19	52	PSSA	Soft tissue	…	3	1	No	0	…
20	52	MSSA	Bacteremia, no focus	…	3	1	Yes	4	Dicloxacillin 1 g QID
21	54	MRSA	Soft tissue	Recurrent/chronic ischiorectal abscess	24	1	Yes	≥12	Ciprofloxacin 500 mg BD + rifampicin 300 mg BD
22	26	MSSA	NV infective endocarditis	…	31	2	No	0	…
23	23	MRSA	Infective endocarditis	…	4	1	Yes	2	Linezolid 600 mg BD + trimethoprim/sulfamethoxazole 160/800 mg BD
24	38	MSSA	Bone/joint	Right shoulder septic arthritis/osteomyelitis. L5/S1 osteomyelitis	15	1	Yes	4	Dicloxacillin 1 g QID
25	41	MRSA	NV Infective endocarditis	…	3	1	No	0	…
26	34	MRSA	Soft tissue	…	9	1	No	0	…
27	46	MSSA	Bacteremia, no focus	…	6	1	Yes	2	Cefalexin 1 g TDS
28	41	MSSA	Bacteremia, no focus	…	1	1	Yes	2	Linezolid 600 mg BD

Abbreviations: BD, 2 times per day; MRSA, methicillin-resistant *Staphylococcus aureus;* MSSA, methicillin-sensitive *Staphylococcus aureus;* NV, native valve; OM, osteomyelitis; PO, by mouth; PSSA, penicillin-susceptible *Staphylococcus aureus;* PV, prosthetic valve; QID, 4 times per day; TDS, 3 times per day.

### Outcome Analysis

Twenty-six of 27 patients had follow-up evident in their medical record, 1 patient had no documented follow-up (direct or indirect) after their index admission and was deemed not clinically evaluable (NCE). Twenty-four of 27 (89%) patients were alive at the end of the 180-day follow-up period, with 2 patient deaths unrelated to their index infection—1 due to ischemic colitis and 1 due to presumed cerebrovascular accident and 1 patient status unknown (NCE). Twenty-six of 27 patients (96%) experienced clinical cure, with 1 patient NCE. Nineteen of 27 patients (70%) did not attend formal outpatient follow-up in either the infectious diseases or other relevant subspecialty clinic; they did however have evidence of indirect follow up through presentations to other network health services documented in their medical record and did not have documented evidence of relapse of infection. Two of 27 patients (7%) had readmissions to hospital within 90 days, both for reasons unrelated to their index infection: 1 from pancolitis, later confirmed as ischemic, and 1 from falls and social concerns.

### Sensitivity Analysis

A binomial 1-sided, 97.5% confidence interval (CI) was calculated using a sample size of 27, where the number of patients who experienced treatment failure equalled 0. The CI for the proportion of recurrences corresponding to zero recurrences observed from a sample of 27 is 0 to 0.127. Therefore, based on our observed population, a true recurrence proportion of more than 12.7% is implausible. Similarly, in the subgroup of 15 patients who did not receive oral antibiotics following administration of the oritavancin, the 97.5% CI for an observed failure of 0, is 0 to 0.21, making a true recurrence rate of more than 20.1% implausible.

### Safety Analysis

Adverse events were categorized into immediate and delayed events. One of 27 patients experienced an immediate adverse event due to peripheral intravenous cannula failure, resulting in localized extravasation. There were no documented episodes of immediate infusion-related reactions, anaphylaxis, or delayed hypersensitivity reactions.

### Cost of Healthcare Analysis

A simplified cost-of-healthcare analysis was conducted. The projected length of treatment for completion of parenteral SOC therapy was 732 days, with a total of 421 hospital days avoided. Hospital days were avoided in 24 of 27 patients (89%) who received inpatient administration of oritavancin before discharge with oral antimicrobial therapy to complete their antimicrobial course (Table 3).

**Table 3.** Outcome Analysis (N = 27)

Primary Outcome	N (%)
Cure	26 (96)
Failure	0
Not clinically evaluable	1
180-d survival	24 (89)
Secondary outcomes—n (%)	
Attended formal follow-up	8 (30)
Lost to formal follow-up	19 (70)
Attended indirect follow-up	18 (67)
Received oral antibiotics following administration of oritavancin	12 (44)
Immediate infusion reaction	0
Delayed infusion reaction	0

Overall, the administration of 30 doses of oritavancin to 27 patients resulted in an estimated cost avoidance to the local health district of $283 100 over a period of 3.5 years. A positive ROI was found for 24 of 27 patients. In 71% (n = 17), the cost avoidance was calculated to be ∼$5000 per patient. The 3 occasions that resulted in a negative ROI occurred in patients who received oritavancin therapy but remained a hospital inpatient long enough to receive the full duration of standard therapy.

The simplified cost-of-healthcare analysis was extended to evaluate the theoretical cost variance between inpatient administration of oritavancin prior to discharge, compared with the projected cost of admission to the local OPAT service for completion of intravenous therapy (Table 4). This analysis assumed all patients in this cohort, who did not complete their intravenous therapy as a hospital inpatient, were assessed and deemed eligible for admission to OPAT for completion of their intravenous antibiotic course. Results of this analysis suggest that the use of oritavancin could be considered cost neutral when compared to OPAT service admission. A cost difference of $12 860 across 27 patients was found, slightly favoring admission to an OPAT service. For 12 of 27 patients (45%), oritavancin therapy was considered cost favorable. For the remaining 55% (15/27) of the patients, the cost of inpatient administration of oritavancin outweighed the projected cost of OPAT admission for completion of the course. When comparing the cost of inpatient administration of oritavancin against the projected cost of admission to an OPAT service (for patients deemed eligible), oritavancin was only considered cost-neutral or cost-beneficial for patients with projected OPAT admissions exceeding >18 days and where a single dose of oritavancin was required for course completion.

**Table 4.** Cost Avoidance Analysis of Oritavancin in Patients with Hospitalized Days Saved

Outcome	N (range)
Mean length of hospital admission	10 d (3–42)
Average hospital day of oritavancin administration	10 d (1–21)
Average hospital days avoided	18 d (6–34)
Mean cost avoidance associated with inpatient oritavancin administration	$13 296 AUD ($600—$31 400)

Abbreviation: AUD, Australian dollar.

## DISCUSSION

This retrospective single-arm cohort analysis describes the clinical outcomes and cost-effectiveness of 27 patients who received oritavancin for the treatment of SAB. Previous studies have demonstrated oritavancin (and other LaLGPs) to be an efficacious alternative for the management of ABSSSIs, resulting in the current US Food and Drug Administration approval for this indication [10–12]. Although off-label use of oritavancin for infections outside of ABSSSIs is being increasingly described, as far as the authors are aware, this is the largest single-arm cohort study looking at the use of oritavancin for the primary indication of SAB.

Our data suggest that oritavancin may be a safe and suitable treatment option for episodes of SAB, including those with foci not limited to ABSSSI and those associated with infective endocarditis, with 96% of patients demonstrating clinical cure. In addition to being a potentially suitable alternative treatment for SAB, our results also indicate that it is a cost-effective alternative to the prolonged inpatient admissions that are often required when patients are ineligible for OPAT. The positive ROI for oritavancin extends beyond the cost saved per patient episode and includes intangible cost benefits of increased hospital bed availability and improved patient flow.

The authors acknowledge these results to be more favorable than those seen in previous studies looking at the use of oritavancin but suggest the following reasons for this result. First, our cohort had a young mean age of 43 years—and likely was less comorbid based on previously described cohorts of PWID [13]. Second, our study was limited to infections due to *S aureus*, and as such, was not subject to the microbiological heterogeneity demonstrated in other studies.

In addition to this being a single-center, single-arm retrospective review, another significant limitation of our study is the use of additional antibiotics before and after the administration of oritavancin. Recently published studies, including that by Wildenthal et al, who looked at the outcomes of partial oral antibiotic treatment for complex SAB in PWID, and the Partial Oral versus Intravenous Antibiotic Treatment of Endocarditis trial, are supportive of a paradigm shift away from the use of prolonged intravenous antibiotics for SAB and infective endocarditis [3, 14]. The mean “pre-oritavancin” duration of intravenous antibiotic administration in our study was 10 days, which is consistent with the minimum intravenous duration used in the aforementioned studies. The authors acknowledge that the stepdown to oral antibiotics after the administration of oritavancin in 12/27 patients, including 4/12 of these patients receiving 1 or more agents considered of high oral bioavailability, has the potential to be a significant confounding variable. Although our study adds to the available evidence, particularly in contrast to the Partial Oral versus Intravenous Antibiotic Treatment of Endocarditis trial by including patients with SAB due to MRSA, further studies are certainly required to confirm the utility of oritavancin over early oral stepdown approaches [14].

A further potential limitation of our study is the significant proportion of patients lost to formalized follow-up and subsequent reliance of indirect measures of clinical success. However, as the patients who did not return for formal follow up did have evidence of ongoing contact with other health providers in the HNELHD, the authors believe that the absence of further presentations concerning for relapse of SAB in these patients is a reliable marker of clinical success. Nonetheless, it may have resulted in underreporting of mild and delayed adverse events following oritavancin administration. Prior studies have suggested an adverse event rate similar to that of vancomycin, with nausea, vomiting, gastrointestinal upset, phlebitis, and infusion-related reactions being the most frequent reported adverse events [10, 15]. Last, the authors recognize small sample size as another limitation of this study; however, they also note the general paucity of research available for the use of LaLGPs for SAB.

Dalbavancin, a lipoglycopeptide similar to oritavancin but with reduced spectrum of activity against Van A *Enterococcus* spp, and with a shorter terminal half-life (147–258 hours vs 393 hours) [16], has demonstrated promising preliminary results for the “line-less” management of SAB in the recent DOTS (Dalbavancin as an option for treatment of *Staphylococcus aureus* Bacteremia) trial. This study, presented at the European Society of Clinical Microbiology and Infectious Diseases Global 2024 Congress, has shown similar clinical efficacy for the use of dalbavancin when compared to the SOC, with a comparable Desirability of Outcome Ranking at day 70 distribution [17]. Our findings are congruent with the DOTS (Dalbavancin as an option for treatment of Staphylococcus Bacteremia) study and adds to the growing evidence for the use of LaLGPs in the management of SAB but has the proposed benefits of single-dose administration due to the longer terminal half-life of oritavancin—a not insignificant potential advantage given the high proportion of loss to follow-up in our population.

## CONCLUSION

Our single-arm observational cohort study has shown oritavancin to be a potentially suitable and safe alternative treatment option for SAB. The demonstrated effectiveness, coupled with its potential for cost-saving and reduced hospital stays, positions it as a valuable alternative for patients with SAB who are ineligible for OPAT. Further randomized controlled trials are required to compare the efficacy of oritavancin to the current standard of care and to confirm its benefit without the concurrent use of additional antimicrobial therapy.

## Supplementary Material

ofaf333_Supplementary_Data

## Supplementary Data

Supplementary materials are available at *Open Forum Infectious Diseases* online. Consisting of data provided by the authors to benefit the reader, the posted materials are not copyedited and are the sole responsibility of the authors, so questions or comments should be addressed to the corresponding author.

## Note

***Potential Conflicts of Interest.*** The authors have no conflicts of interest to declare and did not receive any external funding of incentive to complete this study. All authors: No reported conflicts.
