# Supplementary material for: Oritavancin for the Treatment of Staphylococcus aureus Bacteremia—A Retrospective Single-arm Cohort Study
Source: Open Forum Infect Dis. 2025 Jun 12;12(7):ofaf333. doi: 10.1093/ofid/ofaf333 (PMC12248416; doi:10.1093/ofid/ofaf333)
Supplement: ofaf333_Supplementary_Data [file ofaf333_supplementary_data.docx]

**Appendix 1: Supplementary Information**

Oritavancin for the Treatment of *Staphylococcus aureus* bacteraemia – a retrospective single-arm cohort study.

1: Cost Estimates for Simplified Cost-of-Healthcare Analysis

| Description of Cost | Source |
| --- | --- |
| Cost of admission to an acute care hospital bed | An estimation of cost of admission, as provided by the hospital’s finance officer. |
| Average Wholesale Pricing (AWP) for Oritavancin | The Average Wholesale Price (AWP), as recorded in the hospital's pharmacy inventory management system, rounded to the nearest $500 for convenience and practicality. |
| Cost of admission to an Outpatient Parenteral Antimicrobial Therapy (OPAT) service | An average estimation of cost of admission, as provided by the hospital’s finance officer. |
| Average cost of antimicrobial therapy for patients on OPAT service | The average price of elastomeric infusion devices was estimated based on data from the hospital’s pharmacy inventory management system and rounded to the nearest $10 for simplicity |
